# Moral dilemmas and conflicts concerning patients in a vegetative state/unresponsive wakefulness syndrome: shared or non-shared decision making? A qualitative study of the professional perspective in two moral case deliberations

**DOI:** 10.1186/s12910-018-0247-8

**Published:** 2018-02-22

**Authors:** Conny A. M. F. H. Span-Sluyter, Jan C. M. Lavrijsen, Evert van Leeuwen, Raymond T. C. M. Koopmans

**Affiliations:** 1 Radboud University Medical Centre, department of primary care, Nijmegen, The Netherlands; 2 Novicare, Professionals in Elderly Care, Best, the Netherlands; 3 Radboud University Medical Centre, Nijmegen, The Netherlands; 4 Joachim en Anna, Centre for Specialized Geriatric Care, Nijmegen, The Netherlands

**Keywords:** Vegetative state/ unresponsive wakefulness syndrome, Conflict, Ethics, Family, Moral case deliberation, Shared decision making

## Abstract

**Background:**

Patients in a vegetative state/ unresponsive wakefulness syndrome (VS/UWS) pose ethical dilemmas to those involved. Many conflicts occur between professionals and families of these patients. In the Netherlands physicians are supposed to withdraw life sustaining treatment once recovery is not to be expected. Yet these patients have shown to survive sometimes for decades. The role of the families is thought to be important. The aim of this study was to make an inventory of the professional perspective on conflicts in long-term care of patients in VS/UWS.

**Methods:**

A qualitative study of transcripts on 2 Moral Deliberations (MD’s) in 2 cases of patients in VS/UWS in long-term care facilities.

**Results:**

Six themes emerged: 1) Vision on VS/UWS; 2) Treatment and care plan; 3) Impact on relationships; 4) Feelings/attitude; 5) Communication; 6) Organizational aspects. These themes are related to professionals and to what families had expressed to the professionals.

We found conflicts as well as contradictory feelings and thoughts to be a general feature in 4 of these themes, both in professionals and families. Conflicts were found in several actors: within families concerning all 6 themes, in nurse teams concerning the theme treatment and care plan, and between physicians concerning all 6 themes.

**Conclusions:**

Different visions, different expectations and hope on recovery, deviating goals and contradictory feelings/thoughts in families and professionals can lead to conflicts over a patient with VS/UWS. Key factors to prevent or solve such conflicts are a carefully established diagnosis, clarity upon visions, uniformity in treatment goals and plans, an open and empathic communication, expertise and understanding the importance of contradictory feelings/thoughts.

Management should bridge conflicts and support their staff, by developing expertise, by creating stability and by facilitating medical ethical discourses. Shared compassion for the patient might be a key to gain trust and bridge the differences from non-shared to shared decision making.

## Background

High tech medical treatment can result in survival of patients with severe brain injury and without signs of consciousness in a vegetative state, also called ‘unresponsive wakefulness syndrome’ VS/UWS [1, 2]. Patients in VS/UWS show reflexive behaviour, breathe and open their eyes spontaneously, but show no signs of awareness of themselves or the environment [3]. Recovery of consciousness is unlikely after 12 months following traumatic injuries and 3 to 6 months after non traumatic injuries [4–6].

Brain injury patients in VS/UWS usually pass three phases. In the first, *acute* phase, survival of and stabilization are the therapeutic goals. In the second, *post-acute* phase life, threatening events are less prominent. Then patients might change to another ward or rehabilitation centre, depending on administrative factors, like available facilities, reimbursement policies and different views on the needs for patients in VS/UWS [7]. A recent Dutch study showed that more than 50% of patients in a VS/UWS had not received rehabilitation [8]. In the third, *long-term care* phase, recovery of consciousness fails but the patient becomes medically stable [8]. Then the patient goes to a long-term care facility. Prevalence studies show that patients in VS/UWS might survive for decades, far beyond the prognostic boundaries of recovery [8, 9].

Patients in VS/UWS pose ethical dilemmas to their families and to the professionals involved. Sometimes these dilemmas end up in conflicts. Ethical dilemmas on treatment policies regarding patients in VS/UWS have been described since the 1990s [10]. The cases of Terri Schiavo in the USA [11], Eluana Englaro in Italy [12] and Vincent Lambert in France [13] have been publicly discussed and conflicts came to court. These conflicts focussed on the right to let die versus the right to live as an intrinsic value [14, 15]. The right-to-let-die discussions mostly concerned the right to withdraw artificial nutrition and hydration (ANH). Ultimately, after many years of conflicts and court procedures, the courts approved withdrawal of ANH in these cases.

Whether or not such withdrawal is allowed, depends on national policies and on how lives of patients in VS/UWS are valued [7]. In several countries, physicians are allowed to withhold or withdraw life sustaining treatment (LST) like artificial respiration, resuscitation and ANH when recovery is no longer expected [16, 17]. In other countries, like England and Wales, a court approval is needed, even in case of consensus between the physician and family [18]. Reaching approval can be a lengthy and frustrating process, leading to actual discussions about the contribution of courts [19].

Our study focusses on the Dutch situation, where no court approval is needed to withdraw ANH. The 1990 case of Ineke Stinissen resulted in an ethical, medical and legal framework regarding patients in VS/UWS [5, 6, 20]. Dutch law regards ANH a medical treatment and leaves responsibility on treatment decisions with the physician [21]. The Dutch medical framework considers LST for the sole purpose of prolonging VS/UWS beyond chances of recovery of consciousness to be medically futile [5, 6, 22]. The Royal Dutch Medical Association (KNMG) even stated that prolonging a VS might be seen as ‘a violation of human dignity’ [6]. They also stated: ‘When family of the patient insists on continuation of LST it is the duty of the physician to inform the family and guide them in order to change their opinion, thus physicians being able to withhold LST after consent of the family’ [6]. These recommendations are still valid today.

In Dutch nursing homes Elderly Care Physicians (ECP) are responsible for medical treatment [23]. Between 2000 and 2003, 24/43 patients in VS/UWS in Dutch nursing homes died after a non-treatment decision of an ECP, 9/43 after withdrawal of ANH [9]. Despite the mentioned recommendations for withdrawal, Dutch studies have shown that some patients still receive LST far beyond the period of possible recovery, even beyond 25 years [8, 9, 24]. It is not known why the recommendations to withdraw LST were not followed by physicians. A study on decision making showed that families’ attitudes were crucial in the ultimate decision of the responsible physician [25].

Our study aimed to make an inventory of the professional perspective, on conflicts concerning, patients in VS/UWS in Dutch long-term care settings. Therefore, we did a pilot study on two cases in which disagreement on end-of-life decisions between the ECP and the families resulted in serious conflicts.

## Methods

We present an explorative, qualitative study of the professional perspective in two cases of patients in VS/UWS, residing in Dutch nursing homes, using transcripts on two moral deliberations (MD’s). Both patients survived far beyond the time of possible recovery of consciousness. The MD was requested because of conflicts between the professionals and families. The management of both nursing homes recommended an MD in order to alleviate the burden of the professionals in their daily work and to explore the moral dilemmas they encounter.

An MD is a prospective ethical case deliberation in clinical practice [26]. The aim is to structure multidisciplinary team conferences in situations of future decision making. The MD’s were chaired by an independent ethicist (EvL) and performed according to a 4-step model. Step 1 is the formulation of the moral question at the beginning of the deliberation. Step 2 is an inventory as well as interpretation of clinical details and the patient’s wishes and needs. Step 3 is a normative argumentation and step 4 is a judgment [26].

The participants of the MD were the members of the multidisciplinary team, e.g. nurses, nurse trainees; physicians, speechtherapists, physiotherapists, ergotherapists, social workers, psychologists. Patients and their family did not participate. The first author (CS) notulated the entire MD. The second author (JL) gave an introductory lecture preceding the actual MD about state of the art knowledge on long-term care of patients in VS/UWS and did not participate in the MD. This lecture was followed by a summary of the case history by the treating physician.

In order to have a better notion of the family perspectives we intended to interview the families of the patients. The medical ethical committee had approved the research questions. However, the families refused to participate because of the conflicts.

### Analysis

The hand written verbatim notes of the MD’s were used as a transcript by the first author (CS). The first three authors consented on the transcripts of the MD's. The data were thematically coded and systematically charted, following the principles of framework analysis [27]. The Atlas.ti software program (A6) was used for coding and analyzing.

The first step of the analysis was data reduction. Three analysts (CS, JL and EvL) independently coded the transcripts to minimize subjectivity. Two analysts (CS and JL) are Elderly Care Physicians, experienced in these patients (ECP) and one analyst (EvL) is an ethicist likewise experienced in chairing MD’s. Coding meant that conceptual labels were given to the data. The aim was to attain new insights by breaking through standard ways of thinking about phenomena reflected in the data [28]. The analysts used codes that were strongly related to text fragments, so called quotations. The data about how families had reacted was given by the attendants.

The three analysts compared their codes and discussed them until consensus was reached. A fourth analyst (RK), an ECP, independently reviewed the chosen codes. In the next step, (CS, JL and EvL) grouped codes that referred to the same phenomenon into categories and categories into themes. The themes were discussed with (RK) in a final step until consensus was reached.

The study was judged by the accredited regional medical research ethics committee. According to the Dutch Medical Research Involving Human Subjects Act (1998), the study did not meet criteria for medical scientific interventional research. Therefore no additional ethical evaluation was needed. The committee also stated that since maximal anonymity was secured, consent of the family or the participants of the MD was not required. A consulted independent ethicist confirmed that no formal consent of the families was needed since the families did not participate in the study and anonymity was optimalized. Anonymity was secured by anonymisation of several aspects like the patient’s names, time after incident, age, family relationships and other potentially identifying information, as suggested by Saunders et al. [29].

## Results

Table 1 shows two short summaries of case descriptions as presented by the physicians. Definition of Advance Directive (AD): Oral or written directives from the patient on his subjective whishes and values on treatment decisions [30].

**Table 1:** Medical facts, treatment-plan, Advance Directive (AD), Advance Care Planning (ACP) and family agreement at time of MD

	Case 1 patient A	Case 2 patient B
Duration of VS/UWS	10 years	20 years
Age at time of incident	41 years	17 years
Cause of brain injury	Cardiac arrest	Traffic accident
Medical treatment plan	Tube feeding	Tube feeding is to be withdrawn in time
	Diabetes Mellitus treatment	Seizure control
	Bronchial toilet	Constipation control
	Constipation control	No more physiotherapy
	Physiotherapy	Attempts by social worker, psychologist and mental healthcare to alleviate the burden of the family
Advance Directive by the patient	No prior AD (written or oral)	No prior AD written Reconstructed wish of the patient by the mother: her son would not have accepted to be in a state like this.
End-of-life decisions in ACP	No admission to hospital for any treatment of life threatening complications.	No admission to hospital for any treatment of life threatening complications Maximum of 3 different antibiotics If not successful: palliative care
Family agreement	No, the family demands all treatment including ICU	Ambivalent, possibly the family accept no hospital admission policy, They insist on treatment by antibiotics

Definition of Advance Care Planning (ACP): The process of developing a valid expression of whishes of patients during several meetings [31]. It is a decision making process from the patient, or his family with the treating physician in anticipation of end of life.

‘Family member’ and ‘family’ are purposefully distinguished. The family of a patient is not just one person but consists of a group of individuals. Each individual has its own perspectives, feelings and thoughts, even if the family agrees to one point of view. With ‘family’ we address a family as a whole, with ‘family member’ we refer to one family member in order to see possible differences within a family.

### Case1, patient A

A 41 jears old male was since 10 years in a VS/UWS after a cardiac arrest. The patient resided in a nursing home and didn’t get any kind of rehabilitation before admittance.

He received ANH by tube feeding, had a tracheal tube and a urinary catheter. The medical record mentioned Diabetes and severe breathing disorders. The patient had four times become life-threateningly ill of infections as urosepsis and pneumonia.

The physicians considered life-prolonging treatments as futile. However, due to the pressure of the family, the patient was repeatedly admitted to hospital for a life-threatening event. The family even insisted on intensive care treatment. Many conflicts occurred between the treating physician, nursing staff and the family, mainly about treatment decisions and daily care issues. The family did not consent to participate in a study in which the diagnosis was re-evaluated by an expert.

### Case 2, patient B

The second patient was 17 years old when he was diagnosed in VS/UWS due to brain injury after a traffic accident. At the time of the MD he was in VS/UWS for more than 20 years. He was given ANH by tube feeding and had a urinary catheter. The patient had several bone fractures due to osteoporosis and epileptic seizures. He had not received any kind of rehabilitation.

The many physicians that treated him over the years had no doubt about the absence of signs of consciousness. The diagnosis was more than once established by structured instruments like the Western Neuro Sensory Stimulation Profile and Coma Recovery Scale revised [32, 33].

According to the Dutch medical ethical framework regarding patients who are not to be expected to regain consciousness, the physicians intended to withhold life-prolonging treatment and to withdraw ANH [6]. These intentions were regularly discussed with the family. The parents however, thought there was some kind of consciousness and wanted to prolong his life. Over time many conflicts arose regarding treatment decisions and daily care issues between the family and professionals. During his stay in the nursing home the medical treatment plan on end-of-life decisions was frequently changed.

### Analysis of the transcripts on two moral deliberations

The coding process resulted in 6 themes: 1) Vision on VS/UWS; 2) Treatment and care plan; 3) Impact on relationships; 4) Feelings/attitude; 5) Communication; 6) Organizational aspects. Table 2 shows the codes, categories and themes. The study showed conflicts between families and professionals, within professionals and within families.

1. Vision on VS/UWS

**Table 2:** Themes, related to family and professionals and matching codes and categories

**Vision on VS/UWS**		**Feelings/attitude**	
o Vision family	• Vision family on consciousness - Expectations and hope for future • Disagreement / dispute within family • Inner contradiction/ paradox family	o Emotions in family	• Acceptance/ gratitude • Trust- distrust • Anger/ anxiety/ tension • Negativity • Letting go • Powerlessness • Guild • Concern • Stand up for / Sacrifice
o Vision professionals	• Vision physicians • Opinion paramedics on experience • Vision nurses - Interpretation reactions of patient • Differences in opinion between physicians and in multidisciplinary team - Futile medical treatment	o Feelings and attitude professionals	• Distrusted • Anxiety/ fear of the family • Burnout - Feeling continuously checked - Never being able to do right • Change of board/ profession • Compassion with patient - Compassion with family • Inner contradiction/ paradox professional
**Treatment and care plan**		**Communication**	
o Professional input	• Medical treatment plan - End of life decisions • Nursing treatment plan	o Communication	• Between professional and family • Between nurses and patient • Between professionals
o Wishes family for treatment plan	• Treatment - Everything possible is done • Advance directive family • Inner contradiction/ paradox family	o Making sense and meaning	• Family • Nursing staff
o Actual dealing with treatment plan	• Ignoring agreements • Following agreements • Consultation • Lack of clarity - Crisis situations		
**Impact on relationships**		**Organizational aspects**	
o Family-family o Family-patient	• Disagreement / dispute within family • Impact for family • Involvement, family participation • Conflict - Still the same person as before the accident		• Management • Support • Educational program
o Family-professionals	• Involvement, family participation - Frequent personnel change • Professional support • Conflict		
o Professionals-professionals	• Tension in multidisciplinary team • Frequent personnel change • Support of professional		

(**bold**) Themes

(−) Matching codes

(•) Categories

The theme ‘vision on VS/UWS’ contained the opinions of professionals and families on whether or not the patient had some kind of consciousness, pain perception or awareness of his environment. We found the visions of the physicians were uniform and different from those of some other professionals and from the families.

- Vision of professionals

The physicians of both patients considered them to be unresponsive and unaware of their environment. They were convinced the patients had no pain perception and did not suffer.

The vision of other professionals on VS/UWS in both cases lacked uniformity. One physiotherapist claimed: “When I move the patient’s hand, I see a strong muscle contraction. I interpret this as a sign of pain perception by the patient”. On the contrary, some nurses of the same patient mentioned that they had never seen any sign of pain perception when administering intramuscular injections. These nurses were convinced this patient had no consciousness of himself or his environment. Other nurses were less convinced since they declared the patient showed restless behaviour when his urinary catheter was replaced. However, they saw no apparent restless behaviour when the patient had bone fractures.

- Vision of families

Both families expressed that according to them the patients experienced at least something. The parents of patient B mentioned their son reacted on the presence of his mother. They had told to several professionals: “We feel that he has a deep emotional binding with his mother”. The father was convinced that his son had some kind of awareness. In both cases the professionals mentioned that some siblings had expressed different opinions on their vision of the patient, more in line with those of the physician.

2. Treatment and care plan

In both cases no prior written or oral advance directive (AD) was present. The mother of patient B reconstructed her sons’ wish in that her son would not have accepted to be in a state like this. The cases showed conflicts between the professionals and the families regarding treatment and care plans (Table 2).

- Professionals

In the treatment plans at the time of the MD regular chronic diseases and complications of the VS/UWS were treated. Both care plans mentioned prevention of pressure ulcers. In patient B physiotherapy was cancelled because of lack of recovery. Patient A received physiotherapy upon insistence of the family. Social workers and psychologists offered to support family B in their grief, but they refused.

The physicians of the two patients considered medical treatment to be futile after such a long period of VS/UWS. Therefore, they lacked medical reasons to admit the patient to hospital in case of life-threatening illnesses.

- Family’s wishes for treatment

Both families wished an active treatment in which LST was provided if needed. The family of patient A requested the nurses to check the blood glucose and saturation levels on a daily basis. They also insisted that a physiotherapist moved the joints to prevent contractures. When the patients became seriously ill, the families demanded LST including, in case of patient A, ICU treatment. On the contrary, some family members told if becoming a patient in VS/UWS themselves, they would want LST to be withdrawn.

- Actual dealing with treatment plan

In both patients the different views of the professionals and the families on treatment plans resulted in conflicts. The treatment plans of the two patients were regularly adjusted due to either pressure of the families or to lack of transparency of the plans.

Under pressure of the family of patient A the physician agreed to consult a colleague of the ICU in case of life-threatening illnesses. In shifts, attending physicians mentioned they were not aware of that agreement in the treatment plan.

The physicians in both cases were not uniform in the way they dealt with the treatment plans. Some physicians in case A intended, in the context of advanced care planning, to withhold life-prolonging medical treatments and to withdraw ANH in time. However, after an urgent request of the family another physician did not stick to the plan and prescribed antibiotics to cure pneumonia. After that treatment the family insisted that the patient received antibiotics in case of life-threatening infections. Subsequently the plan was altered. On one event the patient did not recover and the family expected him to die soon and was at peace with it. But another physician ignored the plan and prescribed a third kind of antibiotics and the patient recovered. Since then the family agreed to withhold further life-saving treatments only if three antibiotics would have been tried. Thus the plan was changed again.

When the parents of patient B were informed about the possibility of additional technical diagnostic methods, e.g. neuro imaging, next to standard behavioural assessment, they answered according to the MD: “If consciousness is shown, we have failed in applying the right treatment, activities and leisure. If not… he is still our son”.

The physician in charge of patient B at the moment of the MD mentioned that she “actually treated the parents instead of the patient”. She thought the patient would not be harmed by the treatment, whereas the parents would suffer if their son would die.

3. Impact on relationships

The two families showed a similar pattern of behaviour towards the patients. Most members visited their loved one each day for long periods of time and participated in daily care activities. Some family members, however, did not participate in daily care. Family events were celebrated with the patient as if their relative was the same persons as before. According to the MD, within the families contacts were disrupted because of quarrels regarding the views on VS/UWS and because some family members, e.g. children, felt neglected.

Both families had limited time left to maintain other relationships. The ward and the nurses became more and more their social context.

4. Feelings and attitude

The feelings and attitudes of the families expressed by the professionals in the MD differed from positive feelings of acceptance of the situation and gratitude to negative feelings as distrust and anger (Table 2). Conflicts between family members were mentioned in both cases. Families told the professionals they had to stand up for their relative in order to get adequate care. Professionals observed some inner contradictions within the families. The parents of patient B expressed the fear their son would survive them: “On the one hand they don’t want their son to survive them, on the other hand they demand all possible medical treatment”. The father had mentioned to a physician: “This is a fate worse than death, but I can’t let him go”.

The feelings and attitudes of the professionals differed from positive, compassionate, to negative feelings such as anxiety and fear (Table 2).

In both cases the nurses felt compassion with the patients. The physician of patient A mentioned a high tension between him and the spouse of the patient. The nurses experienced distrust and tried to avoid contact with the family. As one of the nurses mentioned: “I feel a constant surveillance of the family… we can never do it right”. The time the nurses needed to spend on this family had a negative impact on the care for the other patients on the ward; “I can’t give proper care to the other patients this way”. Many nurses had a burnout because of the experienced tension and some even changed from ward or even altered from profession. Nurses felt compassion with the family of B, but some nurses experienced fear: “I fear the father because he had threatened a physician if he would refuse to continue life-sustaining treatments”. Another nurse mentioned: “His father knows where I live …I don’t know what he might do to me if his son dies”. Despite these feelings they felt sorry for the family. They understood their grief and they felt the need to support them.

5. Communication

Communication with the families, as expressed in the MD, was mostly associated with a negative experience. In patient A, the family ignored the decision of the physician in charge and insisted on hospital treatment by calling for an ambulance themselves. Since that incident the treating physician experienced great trouble to communicate with them. The nursing staff of patient A expressed difficulties in communication with the family as they gave too much negative attention. “They always talk negatively about the performance of our job, the clothes are dirty, a knot is missing, he is not sitting straight in his chair, etc”.

Transparency of communication among professionals was mentioned in the MD to be an issue as well. In patient A transparency lacked between physicians, between nurses and between physicians and other professionals. The attending physicians were not informed about the agreement that an ICU physician had to be consulted in case of an emergency. The head nurse had not reported the agreements on family participation in daily care. This poor communication led to tension in the multidisciplinary team. The communication between the professionals of patient B was more transparent and uniform, with less tension within the team.

6. Organizational aspects

The participants of the MD noticed that communication between them and the executive board of the nursing home had changed over time. In the 1990s, the management, with an ECP as an ultimate responsible medical director, was more involved in policy making and treatment scenarios. According to the MD, nowadays the board works on a more distinct level and has less insight in the problems encountered on the ward. The professionals expressed they experienced little support of managers and that educational programs on VS/UWS were lacking.

Frequent personnel changes were mentioned in both cases, regarding physicians, the nurse staff as well as the management. Due to those changes, the professionals had little time to develop and preserve knowledge on such rare and complex patients.

In patient B, 5 different physicians, inexperienced on VS/UWS, were responsible in 1 year. Besides the frequent changes of physicians, this study showed inconsistencies in treatment plans. Family expressed that it was difficult for them to remain trustful because of the frequent changes.

The final step of the analyzing process the data showed inner contradictory or paradoxical feelings or thoughts in the first four themes, both in professionals and families. Table 3 shows some examples of these inner contradictory feelings and thoughts.

**Table 3:** Inner contradictory feelings/thoughts told by the participants of the MD

Theme	Family/professional	Quotation
1. Vision on VS/UWS	Professional	The physiotherapist tells he sees some muscular contractions which he interprets as pain perception while the same patient shows no reactions when administered intramuscular injections
2. Treatment and care plan;	Family	The families when asked declare they would not want this situation for themselves, then life should be ended, yet they wish all possible treatment for their relative
	Family	Mother expresses that she thinks her son would not have wanted this situation in VS/UWS, nevertheless she demands life-prolonging treatment
	family	Expectations and hope for the future: The wife told she knew her husband was unable to regain consciousness, but she demanded physiotherapy for him in order to be able to use his hands when needed
3. Impact on relationships	Family	The mother does not want her son to survive them but on the same time demands life-sustaining treatments
	Family	The father says: “This is a fate worse than death but I cannot let him go.”
	Family	“If consciousness is shown, we have failed in giving the right treatment, activities and leisure. If not.. he is still our son.”
4. Feelings/attitude	Family	Despite the expressed problems with daily care the family expresses they have trust in the nurses
	Family	The wife simultaneously mentions expectations of hope and despair
	Professional	One of the nurses tenderly taking care of the patient since 20 years, expressed “In case of an FMRI investigation, I hope they will not see any sign of consciousness; I cannot imagine what it would be like to live this way for more than 20 years….. if only a fraction of experience would enter the brain, so more reason to stop this situation.”

## Discussion

In this explorative study on ethical dilemmas in long-term care patients in VS/UWS, 6 themes emerged: 1) Vision on VS/UWS; 2) Treatment and care plan; 3) Impact on relationships; 4) Feelings/attitude; 5) Communication; 6) Organizational aspects. These themes are related to professionals and to what families had expressed to the professionals.

We found conflicts as well as contradictory feelings and thoughts to be a general feature in 4 of these themes, both in professionals and families. Conflicts were found in several actors: within families concerning all 6 themes, in nurse teams concerning the theme treatment and care plan, and between physicians concerning all 6 themes.

The discrepancies in vision on VS/UWS between families and physicians, between professionals and within families are key findings of our study. Responses of the patient are regarded as signs of awareness and even pain perception. Such interpretations of responses were also found in other studies, among physicians and physiotherapists [17, 34]. Misinterpretation of signs can easily lead to misdiagnosis, which was repeatedly found to be around 40% [8, 35, 36]. Both, misdiagnosis and the discrepancy in vision on VS/UWS are also a source of conflict.

We found a high involvement of the families in daily care, even after 10 to 20 years, and we found a high impact on relations both within the families and between families and professionals. Studies on post-acute care also showed high involvement of the families in daily care and a great impact on their social relations [37, 38]. Caregivers of patients in severe to moderate traumatic brain injury (TBI) in long-term care often feel they cannot leave the person they care for, like they are stuck and isolated [39]. However, the impact on the relation between the families and the professionals as we found is not described in these studies.

Professionals and families experienced negative feelings, e.g. anxiety, fear and distrust, anger, as well as positive feelings, e.g. compassion and gratitude. Negative feelings in families like denial, guilt and anger have been mentioned in other studies in earlier phases when prognosis is not yet certain [40]. In that phase hostility towards professionals, like in our case, is regarded the result of families’ inability to complete their bereavement process [40]. We know from Italian studies in both post-acute and long-term care of Disorders of Consciousness (DOC) patients that about 30% of caregivers had a progressive grieving disorder [41]. In a study among caregivers of mild to moderate TBI in long-term care, parents express more intense feelings of grief than partners, related to loss of their child as it was before the TBI, the envisioned future, and endorsed guilt and a sense of responsibility for their child’s injury [39]. That study also shows that in many cases, caregivers express anger and frustration related to the challenges of managing daily tasks and interactions with medical providers, family and community members, and the person with TBI [39].

Positive feelings of trust by the families are also mentioned in the post-acute phase [42]. These feelings are considered to be due to the empathetic manner in which information is given and to human and personal contact with the relatives and the patient and to the common goal, survival of the patient [42].

In our study, the families still focussed on survival. However, the treatment goal of the physicians had altered and, as recovery was no longer expected; they intended withdrawal of LST. These differences in goals and focus are a source of conflict. We also found great compassion and empathy of the professionals for their patients and in one case for the family as well. In the other case there seemed to be much distrust. In literature such negative feelings between professionals and family have not been explicitly mentioned, though a high burnout level among professionals is found in studies concerning DOC patients in long-term care [42]. An overall figure of 20% is mentioned and even 25% in the nursing staff [43]. Such high burnout levels might well be the result of the high impact on relations between families and professionals and of negative feelings like distrust.

The physician has a special role in the Netherlands. Unlike in other countries, where court approvals are needed to withdraw ANH in case of VS/UWS, the Dutch physician is in charge to decide what is the best appropriate treatment for the patient after consulting the family. This MD study showed that good communication on vision and treatment goals, is crucial in shared decision making between professionals and families. The information about the diagnosis and the expected prognosis was not always uniformly discussed by the professionals. The relevance of interventions and therapies were scarcely communicated with the family by the responsible physician. The sometimes twisted information, given to the family, contributed to distrust and conflicts. In patient B, diagnosis, prognosis and treatment goals had been discussed by the physician in an early stage. This led to more clarity for other professionals and the family, though the difference in goals of physicians and the families still led to conflicts and distrust.

Not only the content of communication but also the means of communicating proved to be important. Mostly attitude, timing and differences between professionals had a negative connotation and led to more emotional burden and conflicts. However, in periods where communication was more transparent there seemed to be less tension and professionals were able to have more compassion with the family. To our knowledge, communication about these highly complex patients in long-term care has hardly been studied. One study in the acute phase showed that relatives experienced more trust when given accurate information in an empathetic manner [42]. A study in the post-acute phase showed an overall need for information by caregivers regarding the disease [41]. A genuine two way dialogue between practitioners and families about the aims of physiotherapeutic interventions in the post-acute phase of DOC patients proved to be critical for establishing positive relationships [44]. In rehabilitation centres, families are even incorporated in the multidisciplinary teams after they are informed on DOC and after their needs are inventorised [45]. Both in the acute phase and in at least the first part of the revalidation period, the goals of families and physicians are the same. In time professionals change their treatment goals while families might still cling to their hope. This might be one of the reasons of conflicts in long-term care.

Organizational aspects were relevant in both cases: discontinuity in policy of the board and in managers, frequent personnel change on the ward and inconsistencies in medical policy. Instability in management seemed to negatively influence the performances of professionals, as it led to insufficient training and to lack of clarity in medical and nursing records. The frequent personnel change and lack of medical leadership, on both the level of the patient and the organisation, seemed to be key factors in the inconsistencies in the treatment plans and in the arousal of distrust in the families.

In literature, as far as we know, nothing is described about such organizational aspects regarding these patients. However, some long forgotten management reports have emphasized the need for managers to facilitate the medical ethical discourse and to bridge differences concerning these patients [46].

We found inner contradictory feelings in several themes. The non response of one family to an independent assessment and the non response of both families to the offered interviews on their vision may depict an inner contradictory feeling. Although families demanded the best possible treatment and care, they refused to participate in an investigation to confirm the diagnosis in VS/UWS, believing the patient had some level of consciousness. A recent study in the UK on family perspectives showed that families were less interested in the medical diagnosis, distinction between VS/UWS or minimally conscious state (MCS), but more, in the interest of the patient, in whether or not LST should be administered [47]. Inner-contradictory feelings have also been mentioned in case reports. In a personal report a mother of a child in VS/UWS mentioned inner contradictory feelings as she simultaneously wanted any life-threatening medical problem to be aggressively treated while knowing that death might be a liberation for her child [48]. Another example is in a study among care givers in mild to moderate TBI the ‘worry about who will provide care when they are gone’ [39]. Inner contradictory feelings have also been mentioned regarding the conflict about the patient’s identity, either dead or alive, even leading to a worldwide discussion on organ donation of patients in VS/UWS [49]. In order to establish identities of the injured persons, many relatives try to make sense of the situation by finding ways to include them in meaningful social practices, like in our cases by celebrating family events with the patient [50]. The caregivers’ identity is reported to become ambivalent as well. A striking example of such ambivalent identity is the simultane social role of spouse and caretaker [51].

A strength of the study is the use of MD’s in cases with patients in VS/UWS. That is a new approach in long-term care. In these cases conflicts were at stake. Therefore, medical ethical dilemmas were studied in the context of conflicts. This method made it possible to explore each case profoundly. We used transcripts of the MD's instead of interviews of the participants because in an MD one is able to explore differences in interpretations that might lead to differences in behaviour, while in interviews one can only study the facts told by participants.

Another strength is that each individual participant of the MD had an equivalent opportunity for input [26]. We have no indication that the structure of the MD limited what participants thought they could say. A strength of a MD is that all participants have an equal input and can address their feelings and opinions from their own perspective. This is stressed in the beginning of the MD by the independent ethicist. In these MD's the ethicist gave equal opportunity to all participants to express themselves freely. The ethicist created a safe atmosphere and encouraged all members of the MD to share their knowledge, ideas and opinions. The wide range of facts, including, deviating facts and opinions, that were put forward underline the save atmosphere and freedom to speak. That is why the facts were given in a broad sense without bias regarding specific professionals. Although this MD study is about just two cases, they represent a significant sample of the whole population of VS/UWS patients in Dutch long-term care. In a systematic review of prevalence studies of VS/UWS, the Netherlands had the lowest (0.2/100000) of the world [52]. And in two nationwide prevalence studies of patients in VS/UWS in 2003 and 2012, respectively only 5 and 3 patients were found in long-term care beyond 10 years [8, 9, 24].

A limitation of this study is that the notes of the MD’s were handwritten, not audiotaped, because of privacy aspects. Audio taping of the MD might have been a better method but we do not think it introduced a bias in themes as the first three authors all observed or chaired the MD and agreed on the notes. They had consented on the transcripts of the MD's. After the transcripts were written they independently coded the transcript and finally they had consensus over the themes.

Another limitation is that the information concerning the families was given by the professionals. This was the only possibility left because the conflicts made the families refuse to tell their views and story. The lack of direct information from them might result in a bias regarding the information of families, as professionals tell what they think families think. We think here three different aspects are at stake: Firstly, the information on what the families communicated seems quit reliable since multiple sources/ professionals in the MD -nurses, psychologists, social workers, physicians, speech therapists and physiotherapists- mentioned what the family told them. Sometimes these expressions were quoted literally, as the members stressed in the MD that they paraphrased the family. Secondly, we acknowledge a possibility that the professionals expressed their own perspectives and even frustrations, instead of those of the families. These perspectives and even frustrations might also contribute to conflicts. The ethicist that chaired the MD's made sure that all aspects concerning the families were sincerely discussed by everyone involved. Thirdly, in the MD the participants mentioned how they thought the families thought and felt. Although this is indirect information we still think that it is important to understand how care providers reflect and interpret how the families think and feel. Given the prolonged contacts between the professionals and the families conflicts are likely to happen as the patients have been treated many years and the families visit their relative daily and participate in daily care. The way professionals experience families' perspectives might contribute to the conflicts.

Recommendations for medical practice, investigations en education.

Especially in the complex situation as VS/UWS, an Advance Directive (AD) might give direction to the wishes and values of the patient regarding end-of-life decisions. In that way an AD can prevent conflicts regarding the subjective wishes and values of the patient as described in these cases, as it depicts the autonomy of the patient. If no AD is available the physician has to make the critical medical decision after consulting the family about treatment for the patient.

Given the complexity and the small numbers of these patients and the high percentage of misdiagnosis adequate expertise, including on the use of CRS-r is, absolutely a necessity.

We recommend an expert to assist in establishing the diagnosis, prognosis and subsequent treatment goals and plans. And since the complexity of medical ethical dilemmas we propose the introduction of an MD. In an MD, led by an independent ethicist, experts and all professionals involved in a particular case discuss the ethical dilemmas. In the MD aspects like diagnosis, prognosis, treatment goals and plans but also aspects like views of the families on VS/UWS, their hope on recovery, and impact on relationships should be considered.

We recommend education of all professionals regarding: diagnosis, prognosis, appropriate treatment plans and medical ethical considerations of patients in VS/UWS; excellent communication skills, insight in the effects of inner-contradictory feelings and thoughts.

We also recommend organisations dealing with these patients to provide stability in the teams and support their personnel, also in facilitating MD’s during the process [46].

For the patients these recommendations might result in the most appropriate treatment. For the families support by experts, more uniformity in diagnosis, prognosis and treatment goals might prevent conflicts. Through such an approach court procedures to allow withdrawal of ANH, like recently in England, often considered time consuming and frustrating for the families involved, might no longer be necessary [19]. For the professionals the above mentioned recommendations can facilitate their work.

We recommend for future studies, in-depth interviews with families and with physicians further exploring their role in the decision making process and in dilemmas. With a special focus on the themes and contradictory feelings and thoughts found in this study. We have already performed such interviews with all the Elderly Care Physicians (ECP's) of a cohort of all patients in a VS/UWS in the Dutch nursing homes. Currently we are analyzing these data. We have started interviewing families of other patients in VS/UWS using the themes found in this study in the actual interview guide.

## Conclusion

Different visions, different expectations and hope on recovery, deviating goals and contradictory feelings/thoughts by families and professionals in patients with VS/UWS can lead to conflicts. Key factors to prevent or solve such conflicts are a carefully established diagnosis, clarity upon visions, uniformity in treatment goals and plans, an open and empathic communication, expertise and understanding the importance of contradictory feelings/thoughts.

Management should bridge conflicts and support their staff, by developing expertise, by creating stability and by facilitating medical ethical discourses. Shared compassion for the patient might be a key to gain trust and bridge the differences from non-shared to shared decision making.

## Abbreviations

ACP: Advance Care Plan
AD: Advance Directive
ANH: Artificial Nutrition and Hydration
DOC: Disorders of Conciousness
ECP: Elderly Care Physician
KNMG: The Royal Dutch Medical Association
LST: Life Sustaining Treatment
MCS: Minimally Conscious State
MD: Moral Deliberations
TBI: Traumatic Brain Injury
VS/UWS: Vegetative State or Unresponsive Wakefulness Syndrome
